# Knowledge and attitude towards COVID-19 and its prevention in selected ten towns of SNNP Region, Ethiopia: Cross-sectional survey

**DOI:** 10.1371/journal.pone.0255884

**Published:** 2021-08-06

**Authors:** Misganu Endriyas, Aknaw Kawza, Abraham Alano, Mamush Hussen, Emebet Mekonnen, Teka Samuel, Mekonnen Shiferaw, Sinafikish Ayele, Temesgen Kelaye, Tebeje Misganaw, Endashaw Shibru

**Affiliations:** 1 Health Research and Technology Transfer Directorate, SNNPR Health Bureau, Hawassa, Sidama, Ethiopia; 2 SNNPR Health Bureau, Hawassa, Sidama, Ethiopia; 3 SNNPR Policy Study and Research Institute, SNNPR President Office, Hawassa, Sidama, Ethiopia; 4 Public Health Institute Director Office, SNNPR Health Bureau, Hawassa, Sidama, Ethiopia; Jouf University, Kingdom of Saudi Arabia, SAUDI ARABIA

## Abstract

**Background:**

COVID-19 is highly infectious viral disease that can lead to main clinical symptoms like fever, dry cough, fatigue, myalgia, and dyspnea. Since there is no drug to cure the disease, focusing on improving community awareness related to prevention methods is crucial. But there was no regional level study addressing the reach of information, community knowledge and attitude related to COVID-19 and its prevention, and this study was done to inform and assist communication related to the disease responses during early introduction of the disease to the setting.

**Methods:**

Community based cross sectional study was conducted in selected ten towns of SNNPR, Ethiopia. Multi-stage sampling was used to select 1239 participants. Semi-structured questionnaire was designed, pre-tested and uploaded to SurveyCTO data collection system with security patterns. Knowledge was assessed considering awareness about signs and symptoms, confirmatory test (laboratory test), what to do if there is a suspect, availability of drug to cure the disease, mechanisms of transmission, prevention methods and most at risk groups. Attitude was assessed using 11 statements including seriousness of disease, being at risk, possibility of prevention, and benefits of staying at health facilities. Descriptive statistics and binary logistic regression were performed to manage data using SPSS version 25.

**Results:**

Almost all respondents (99.8%) heard about the disease. The mean score of knowledge was 52.3% (SD = 18.9) while the mean score attitude was 80.8% (SD = 6.48). Educational status, housing condition and marital status were associated with having good knowledge while occupation, housing condition, age and overall knowledge were associated with having positive attitude.

**Conclusion:**

Even though almost all respondents had heard about the COVID-19, knowledge and attitude related to COVID-19 and its prevention were low. Awareness creation should be intensified using different local languages to improve community awareness, overcome misconceptions and minimize consequences of the disease.

## Background

Coronaviruses are a large family of viruses that can cause a range of illnesses in humans, from the common cold to severe acute respiratory syndrome (SARS) [1]. Coronavirus disease 2019 (COVID-19) is highly infectious disease, and its main clinical symptoms include fever, dry cough, fatigue, myalgia, and dyspnea [2].

Since the World Health Organization’s (WHO) declaration of COVID-19 as a pandemic, many efforts have been carried out to contain the virus [3]. But COVID-19 pandemic continues to cause huge social and economic impacts and stress on the healthcare system of all countries in the world [4, 5].

The WHO warned Africa for COVID-19 pandemic because of challenging conditions like high population density and lack of running water, and also presence of non-specific symptoms of COVID-19 that make it difficult to differentiate from endemic illnesses such as malaria and influenza [6]. In urban settings, there is high risk of transmission of the disease because of dense population, small informal dwellings, lack of access to clean water, presence of multi-generational households with shared sanitation facilities, high level of social mixing, and transient residents [7].

In Ethiopia, the first case of COVID-19 was registered in March, 2020. Since then, the ministry of health warned all regions to be ready to respond, both for prevention and control activities. As part of the responses, the regional health bureau of Southern Nations, Nationalities and People’s Region (SNNPR) in collaboration with partners conducted advocacies and social mobilizations through various methods to increase community awareness [3].

In addition, after the introduction of the disease to the region, the region prepared different quarantine, isolation and treatment centers to cut the disease transmission. But due to different psycho-social reasons, people were escaping from prepared centers, refused to be isolated and even to be tested. So, considering these points, this study was carried out to assess knowledge and attitude of the community and thereby to inform the taskforce and stakeholders during early introduction of the disease to the region.

## Methods

Community based cross sectional study was conducted in selected ten towns of SNNPR from May 18 to June 10, 2020. SNNPR was the third largest region representing nearly 20% population of the country. It is the most diverse region in the country having more than 56 ethnic groups and languages as well as cultures. The region was administratively sub divided in to 18 zones, one city administration and seven special woredas. Currently, the SNNPR region is divided in to two administrative regions, Sidama and SNNPR.

Sample size was estimated by using a single population proportion formula at 95% confidence level assuming proportion of population with good knowledge to be 50% and considering 5% margin of error and design effect of 3. The final sample size was 1268 after adding 10% non-response rate.

Multi-stage sampling was used to select study participants. First, ten towns were purposely selected based on the potential risk of COVID-19 in the region because of crowdedness and high population mobility. At the second stage, two kebeles from each town were selected. Kebles are the smallest administrative units in the study setting. The calculated sample size was allocated to towns based on the size of the urban population. At the time of data collection, there was state of emergency but it was not strict and some people were moving in towns. To include population staying at home and those moving in the town, one-third of sample size was allocated to population walking in towns while two-thirds were allocated to population staying at home.

Kebeles in the towns were listed and random sample kebeles were selected from each town. Data collectors went to the center of selected kebeles and spin pen to select the direction and first house. The next household was selected from next adjacent block systematically and data collection continued until sample size allocated to population staying at home was fulfilled. Regarding population walking, every other person that the data collectors met while moving to the next block were selected and interviewed until sample size allocated to population walking was fulfilled. The interval for systematic selection of walking individuals was minimized because of the assumption that few people may move in towns because of state of emergency. Individuals residing in the selected kebeles at least for six months and age above 18 were considered eligible.

Semi-structured questionnaire was prepared by reviewing relevant literatures [7–9]. The prepared tool was shared with Hawassa University Scientific Advisory Committee (SAC) and comments were incorporated. Face and content validities were assessed by sharing final tool with regional level emergency operation center (EOC) that comprises experts from different international and local non-governmental organizations, Hawassa University, regional health bureau and technical advisory team (TAT) to check if tools can capture required data. The questionnaire was initially prepared in English, and then translated to Amharic. The translated version was again translated back to English language by different experts to check consistencies in the meaning of words and/or concepts. The questionnaire was pre-tested in similar towns but not included in actual study. The data collection tool was prepared with security patterns (must enter and skip commands) and uploaded to SurveyCTO data collection system. Training with field exercise was given to data collectors to have common understanding on data collection tool and process. Keeping COVID-19 prevention measures, face-to-face interview was conducted by nine data collectors who had BSc degree and above and experience in data collection. Field supervision and online monitoring were done daily to maintain data quality.

S1 File data was downloaded from SurveyCTO server and exported to SPSS for Windows version 25 for data management. Descriptive analysis was done to describe study participants and other variables and outputs were presented using frequency, percentage, means and standard deviations and displayed using tables and graphs. Knowledge questions were recoded to “yes” based on fulfilment of specific criteria (Table 6). For example, respondents’ knowledge about signs and symptoms was accepted if respondents answered fever, cough and difficulties in breathing as signs and symptoms. Finally, scores of knowledge and attitude were summed and mean (in percent) with standard deviation were presented. Both knowledge and attitude scores were categorized in to two groups based on mean score. Categories were recoded good or positive if score was equal to or above mean, and scores below mean were recoded poor or negative. Factors associated with good knowledge and positive attitude were assessed using binary logistic regression. Variables with p-value of less than or equal to 0.20 during bivariate analysis were considered for multivariate analysis as used in similar studies [10–12]. Finally, variables with p-value less than 0.05 during multivariate analysis were reported as associated factors with adjusted odds ratio (AOR) and 95% confidence interval.

Ethical clearance was obtained from the Ethical Review Committee of Southern Nations Nationalities and People’s Regional Health Bureau. Official support letters was written to each study towns. Verbal consent was approved (because of its popularity in the study setting) and obtained from study participants. All collected data are kept confidential.

## Results

A total of 1239 participants were included in the study, with overall response rate of 97.7%. From 1239 respondents interviewed, 833 (67.2%) were approached at home while 406 (32.8%) were approached while walking in the town.

### Socio-demographic characteristics of respondents

About half, 657 (53%), of respondents were females and about two thirds, 795 (64.2%), were married (Table 1).

**Table 1:** Socio-demographic characteristics of respondents.

Variables	Category	Frequency	Percent
Sex	Male	582	47.0
	Female	657	53.0
Age	≤ 25	304	24.5
	26–30	214	17.3
	31–35	145	11.7
	36–40	148	11.9
	41–45	74	6.0
	46+	213	17.2
	I don’t know	141	11.4
Marital status	Single	370	29.9
	Married	795	64.2
	Divorced	36	2.9
	Widowed	31	2.5
	Living separately	7	.6
Educational status	No formal education	126	10.2
	Primary (1–8)	249	20.1
	Secondary (9–12)	375	30.3
	Certificate and above	489	39.5
Occupation	Student	172	13.9
	Farmer	29	2.3
	Merchant	315	25.4
	Employee	340	27.4
	House wife	208	16.8
	Daily laborer	42	3.4
	Pensioner	30	2.4
	Private	52	4.2
	Other	51	4.1
Family size	≤ 5	858	69.2
	≥ 6	381	30.8
Religion	Orthodox	475	38.3
	Protestant	594	47.9
	Muslim	137	11.1
	Others	33	2.7

### Sources of information

**Fig 1:**
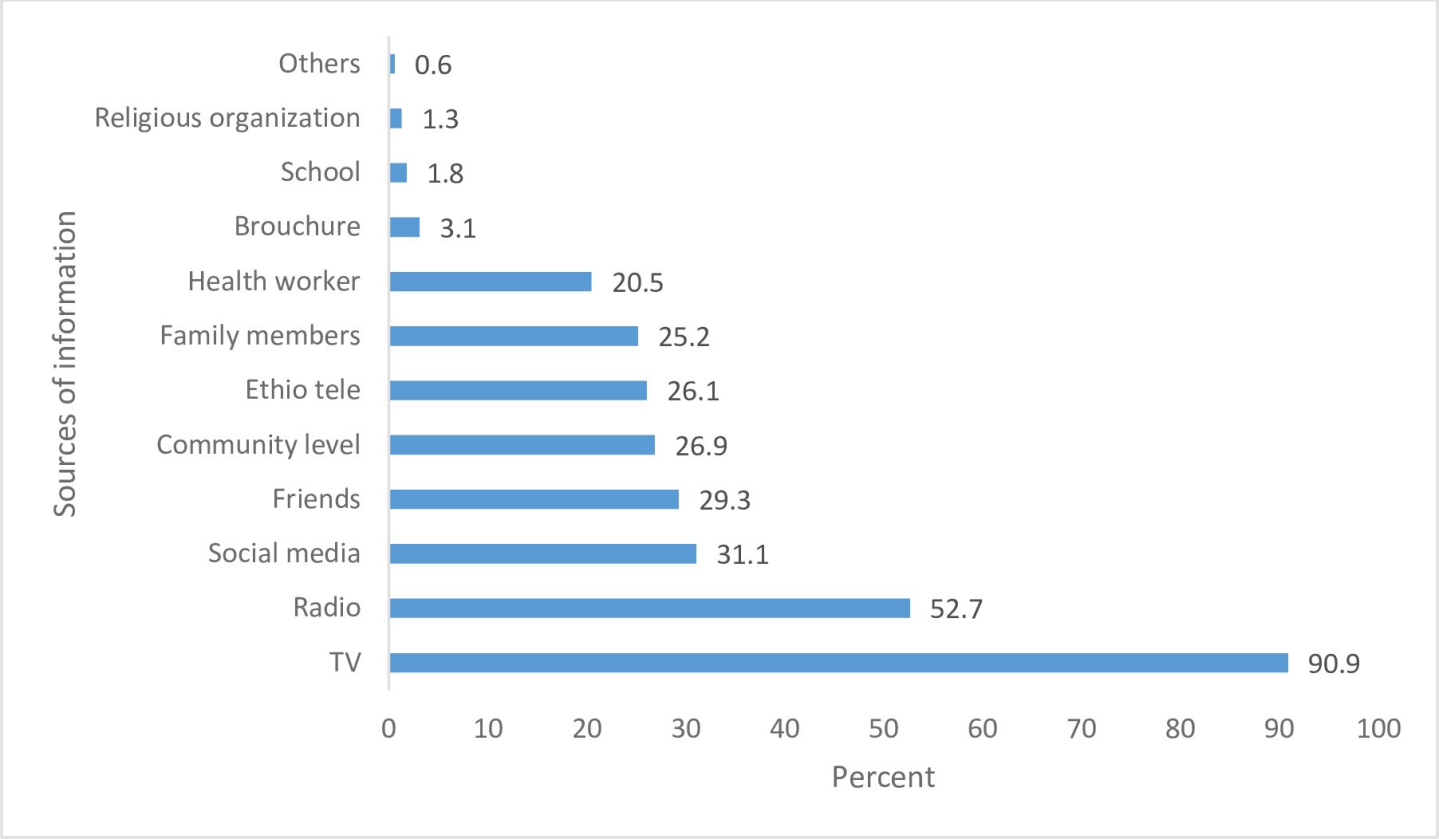
Sources of information about COVID-19.

Almost all, 1236 (99.8%), respondents heard about COVID-19 within two months of introduction of the disease to the country. Majority (90.9%) of respondents heard about COVID-19 from TV, followed by radio (52.7%) and social media (31.1%) (Fig 1).

### Knowledge about COVID-19 and its prevention

#### A. Signs and symptoms

**Fig 2:**
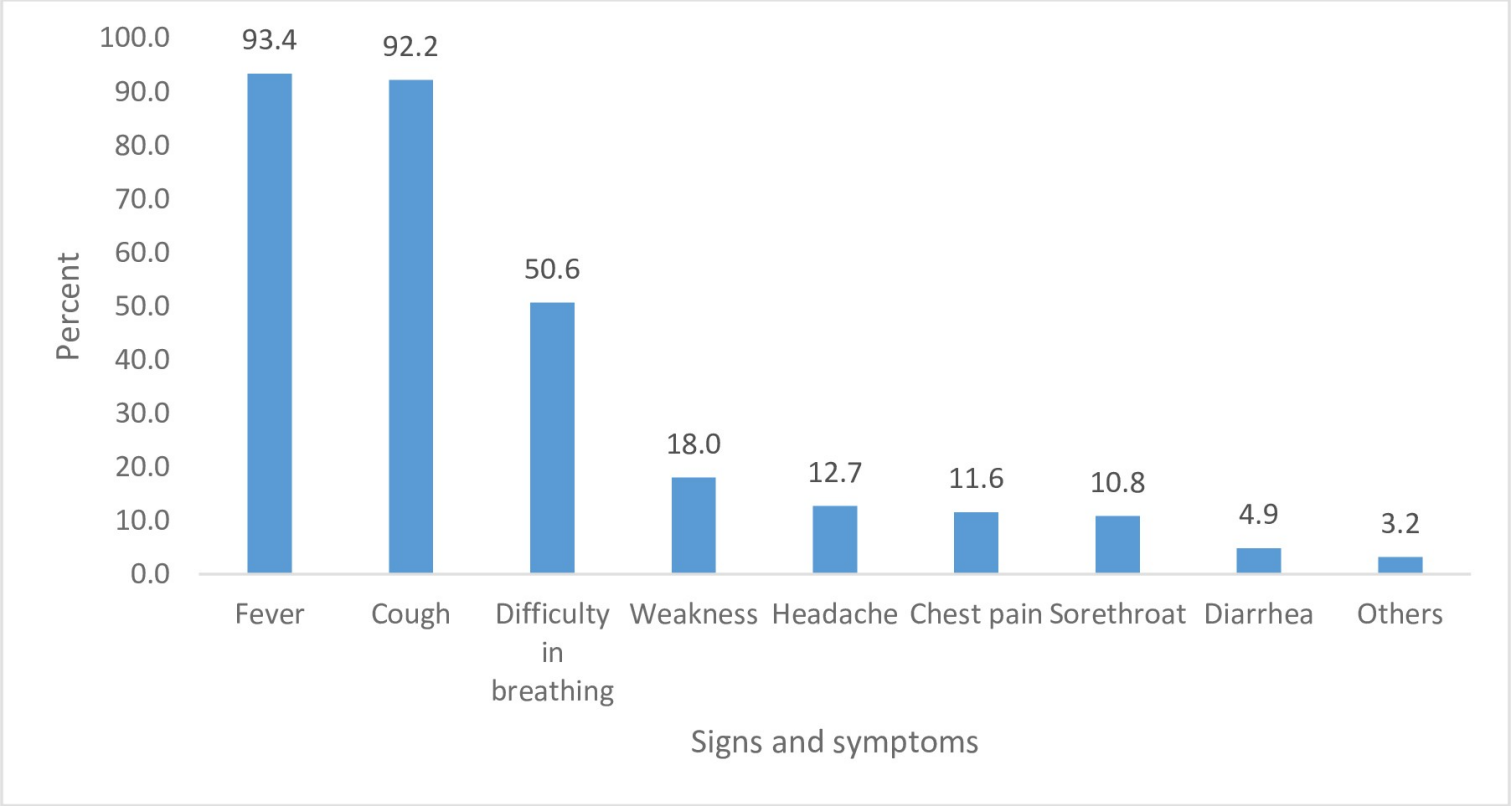
Signs and symptoms reported by respondents.

From those who have heard about COVID-19, majority (93.4%) reported fever and nearly the same amount (92.2%) reported cough as signs and symptoms of COVID-19 while difficulties in breathing was reported only by about half (50.6%) of respondents. Other symptoms like weakness, headache, chest pain, sore throat and diarrhea were reported only by less than two fifths of respondents (Fig 2).

#### B. Confirmatory test

Respondents were asked about confirmatory test of COVID-19, that is how to confirm if a person has COVID-19 or not. About four fifths (81.0%) mentioned signs and symptoms while only two fifths (39. 7%) mentioned laboratory test. Very few reported that it is not possible to confirm (2.1%) and not sure of test (0.7%).

#### C. What to do if there is a suspect

Respondents were asked to report what to do if they face a suspected case of COVID-19. Majority (96.1%) said that they will report to health facilities while 21.5% said that they will quarantine at home. Even though few respondents stated it, misconceptions and fears like “I will shout” were reported (Table 2).

**Table 2:** What to do if there is suspect.

What to do if there is suspect	Frequency	Percent
Report to health facility	1188	96.1
Quarantine at home	266	21.5
Call to hotlines	55	4.4
Give traditional medicines/spices	17	1.4
Report to police	15	1.2
Others*	25	2.0

Others

*—take to holy water, give mask, take to religious organization, give anointing oil, pray, shout

#### D. Drugs to cure

Majority of respondents, 1201 (97.2%), reported that there was no drug to cure COVID-19 while 35 (2.8%) reported that there was drug to cure and mentioned modern drugs like chloroquine (15 people), traditional medicine like spices (13 people) and religious things like holy water and anointing oil (11 people).

#### E. Transmission

Almost all, 1218 (98.5%), respondents reported that COVID-19 is transmittable and majority (91.7%) reported being in close contact (less than 2m) with infected person as mechanism of transmission (Table 3).

**Table 3:** Mechanisms of transmission.

Mechanism of transmission	Frequency	Percent
Being in close contact (<2m) with infected person	1134	91.7
Touching contaminated utensils and greeting unclean hands	1076	87.1
Touching face with unclean hand	642	51.9
Others*	15	1.2

*- Contact with animal, eating raw foods, sexual intercourse, sharing latrine, sweat and transplacental

#### F. Ways to prevent COVID-19 transmission

Nearly nine out of ten, 87.4% and 87.7%, respondents reported social distancing and keeping hands clean as COVID-19 prevention methods respectively while wearing mask was reported by about only one-third (36.8%) of respondents (Table 4).

**Table 4:** Ways to prevent COVID-19 transmission.

Prevention methods	Frequency	Percent
Social distancing	1080	87.4
Keeping hands clean	1084	87.7
Not touching face with unclean hands	618	50.0
Wearing masks	455	36.8
Not greeting/touching contaminated things	65	5.3
Wearing gloves	58	4.7
Others*	13	1.1

*- Praying, taking hot drinks, impossible

#### G. Most at risk population

About two thirds (67.2%) and about half (54.2%) of respondents reported old ages and patients with underlying sickness as most at risk groups for COVID-19 respectively. About two-fifths (40.6%) reported highly exposed groups like health workers, mobile groups and caregivers (Table 5).

**Table 5:** Most at risk population.

Risk group	Frequency	Percent
Old ages	830	67.2
Patients with underlying sickness	670	54.2
Exposed (health workers, mobile groups, caregivers)	502	40.6
Children	119	9.6
Pregnant	29	2.3
Street dwellers	39	3.2
Poor	36	2.9
Everyone	32	2.6
Addicted people	25	2.0
Developed (white people)	20	1.6
Others*	25	2.0

Others

*- males, rural area, young people

### Knowledge summary

In summary, each of knowledge questions were recoded into “yes” based on fulfillment of basic points that should be addressed. For instance, knowledge about signs and symptoms was accepted (recoded as yes) if a person answered at least fever, cough and difficulties in breathing that are very common symptoms of COVID-19. Accordingly, less than half (45.3%) clearly reported these three signs and symptoms (Table 6). Finally, the mean score of knowledge was 52.3% (SD = 18.9).

**Table 6:** Knowledge summary.

Knowledge questions	Frequency	Percent
Know signs and symptoms (fever, cough and difficulty in breathing)	560	45.3
Know confirmation (lab test)	491	39.7
Know what to do if there is suspect (reporting to health facility)	1188	96.1
Know that there was no drug to cure	1201	97.2
Know transmission (touching face with contaminated hand, being within 2m contact with infected patients and touching contaminated utensils)	524	42.4
Know prevention (social distancing, clean hands and wearing mask)	327	26.5
Know most at risk groups (old age, people with underlying sickness and exposed groups)	234	18.9

### Factors associated with knowledge about COVID-19 and its prevention

About half, 646 (52.1%), of respondents scored above mean and recoded as good knowledge. During bivariate analysis, all the seven independent variables had p-value of less than 0.20 and were considered for multivariate analysis. But during multivariate analysis, only educational status, housing condition and marital status had p-value less than 0.05. “I don’t know” category of age and “others” category of occupation had association but overlooked because of their non-specificity (Table 7).

**Table 7:** Binary logistic regression of knowledge about COVID-19 and its prevention.

Variable	Category	Knowledge about COVID-19 and its prevention		COR 95%CI	AOR 95%CI^a^
		Poor	Good		
Sex	Male	266	316	1	
	Female	324	330	0.86 [0.68–1.07]	
Marital status	Single	162	208	1	
	Married	394	400	0.79 [0.62–1.01]	0.69 [0.49–0.99]
	Others	34	38	0.87 [0.52–1.44]	
Educational status	No formal education	83	40	1	
	Primary (1–8)	137	112	1.70 [1.08–2.67]	
	Secondary (9–12)	187	188	2.09 [1.36–3.20]	2.09 [1.27–3.44]
	Certificate and above	183	306	3.47 [2.28–5.28]	3.43 [2.01–5.83]
Occupation	Student	93	79	1	
	Farmer	21	7	0.39 [0.16–0.97]	
	Merchant	156	159	1.20 [0.83–1.74]	
	Employed	129	211	1.93 [1.33–2.79]	
	House wife	109	97	1.05 [0.70–1.57]	
	Daily laborer	25	17	0.80 [0.40–1.59]	
	Pensioner	16	14	1.03 [0.47–2.24]	
	Private	28	24	1.01 [0.54–1.88]	
	Other	13	38	3.44 [1.71–6.91]	3.53 [1.63–7.67]
Housing condition	Private	331	309	1	
	Rent	225	265	1.26 [0.99–1.60]	2.57 [1.38–4.81]
	Flatmate	18	40	2.38 [1.34–4.24]	
	Other	16	32	2.14 [1.15–3.98]	
Age category	≤ 25	170	134	1	
	26–30	100	114	1.45 [1.02–2.06]	
	31–35	70	75	1.36 [0.91–2.02]	
	36–40	68	79	1.47 [0.99–2.19]	
	41–45	42	32	0.97 [0.58–1.61]	
	46+	114	97	1.08 [0.76–1.54]	
	I don’t know	26	115	5.61 [3.46–9.08]	5.71 [3.36–9.70]
Family size	≤ 5	398	458	1	
	≥ 6	192	188	0.85 [0.67–1.08]	

^a^–Non-significant cells are left empty to simplify table

Married respondents were 31% less likely to have good knowledge as compared to single respondents (AOR and 95% CI: 0.69 [0.49–0.99]). Those respondents who attended secondary high school and who have certificate and above were 2.09 times (AOR and 95% CI: 2.09 [1.27–3.44]) and 3.43 times (AOR and 95% CI: 3.43 [2.01–5.83]) more likely to have good knowledge than respondents who did not attend formal education respectively. Moreover, respondents living in rental house were 2.57 times (AOR and 95% CI: 2.57 [1.38–4.81]) more likely to have good knowledge as compared to respondents living in private home.

### Attitude towards COVID-19 and its prevention

Eleven different attitude statements were given to respondents and their agreement to statements was measured using Likert’s scale out of five, from strongly disagree to strongly agree. Majority of respondents agreed positively to the statements (Table 8) with mean score of 80.8% (SD = 6.48).

**Table 8:** Agreements to attitude statements.

Attitude statements	Strongly disagree	Disagree	Neutral	Agree	Strongly agree
I think COVID-19 is serious disease	2 (0.2)	33 (2.7)	8 (0.6)	470 (38)	723 (58.5)
I think that I may get infected with COVID-19	7 (0.6)	118 (9.5)	88 (7.1)	771 (7.1)	252 (20.4)
I think that my family member/s may get infected with COVID-19	7 (0.6)	94 (7.6)	103 (8.3)	824 (66.5)	208 (16.8)
I fear to go to crowded places	0 (0.0)	30 (2.4)	11 (0.9)	730 (59.1)	465 (37.6)
If I take care, I think I can prevent COVID-19	1 (0.1)	10 (0.8)	37 (3.0)	782 (63.3)	406 (32.8)
If people take care, I think it is possible to prevent COVID-19	1 (0.1)	21 (1.7)	43 (3.5)	892 (72.2)	279 (22.6)
I don’t believe that COVID-19 patient can be cured if they get care from health facilities	119 (9.6)	648 (52.4)	139 (11.2)	202 (16.3)	128 (10.4)
I think there is no benefit of taking a person with COVID-19 symptoms and signs to health facility	154 (12.5)	725 (58.7)	31 (2.5)	150 (12.1)	176 (14.2)
I think we can prevent COVID-19 if we keep our hands clean	6 (0.5)	69 (5.6)	62 (5.0)	877 (71.0)	222 (18.0)
I think corona cases should stay at COVID-19 treatment center	1 (0.1)	10 (0.8)	11 (0.9)	820 (66.3)	394 (31.9)
If I get corona, I think I would stay at COVID-19 treatment center	2 (0.2)	13 (1.1)	20 (1.6)	808 (65.4)	393 (31.8)

### Factors associated with attitude towards COVID-19 and its prevention

Attitude of respondents was summarized similarly as that of knowledge level and about half, 603 (48.7%), respondents scored above mean and recoded as positive attitude. During bivariate analysis, as seen in the case of knowledge, all eight independent variables had p-value of less than 0.20 and were considered for multivariate analysis. After running multivariate analysis, occupation, housing condition, age and overall knowledge had p-value less than 0.05 (Table 9). As seen in the case of knowledge, “others” category of marital status had weak association but overlooked because of its non-specificity (Table 9).

**Table 9:** Binary logistic regression of attitude towards COVID-19 and its prevention.

Variable	Category	Attitude towards COVID-19 and its prevention		COR 95%CI	AOR 95%CI^b^
		Negative	Positive		
Sex	Male	277	305	1	
	Female	356	298	0.76 [0.61–0.95]	
Marital status	Single	186	184	1	
	Married	401	393	0.99 [0.77–1.27]	
	Others	46	26	0.57 [0.34–0.96]	0.46 [0.25–0.84]
Educational status	No formal education	59	64	1	
	Primary (1–8)	149	100	0.62 [0.40–0.96]	
	Secondary (9–12)	193	182	0.87 [0.58–1.31]	
	Certificate and above	232	257	1.02 [0.69–1.52]	
Occupation	Student	92	80	1	
	Farmer	3	25	9.58 [2.79–32.93]	9.78 [2.59–36.87]
	Merchant	155	160	1.19 [0.82–1.72]	
	Employed	166	174	1.20 [0.84–1.74]	
	House wife	125	81	0.75 [0.50–1.12]	
	Daily laborer	21	21	1.15 [0.59–2.26]	
	Pensioner	18	12	0.77 [0.35–1.69]	
	Private	30	22	0.84 [0.45–1.58]	
	Other	23	28	1.40 [0.75–2.62]	
Housing condition	Private	305	335	1	
	Rent	261	229	0.78 [0.63–1.01]	
	Flatmate	36	22	0.56 [0.32–0.97]	0.49 [0.27–0.88]
	Other	31	17	0.50 [0.27–0.92]	
Age category	≤ 25	165	139	1	
	26–30	103	111	1.28 [0.90–1.82]	
	31–35	70	75	1.27 [0.86–1.89]	
	36–40	74	73	1.17 [0.79–1.74]	
	41–45	32	42	1.56 [0.93–2.60]	
	46+	93	118	1.51 [1.06–2.14]	2.07 [1.27–3.36]
	I don’t know	96	45	0.55 [0.37–0.85]	
Family size	≤ 5	430	426	1	
	≥ 6	203	177	0.88 [0.69–1.12]	
Overall knowledge	Poor	313	277	1	
	Good	320	326	1.15 [0.92–1.44]	1.29 [1.01–1.64]

^b^–Non-significant cells are left empty to simplify table

Respondents living with others (flatmate) were 51% (AOR and 95% CI: 0.49 [0.27–0.88]) less likely to have positive attitude as compared to respondents living in private houses. Farmers were 9.78 times (AOR and 95% CI: 9.78 [2.59–36.87]) more likely to have positive attitude as compared to students, and older ages (46 and above) were 2.07 times (AOR and 95% CI: 2.07 [1.27–3.36]) more likely to have positive attitude as compared to younger ages (≤ 25). Moreover, respondents with good knowledge were 29% more likely (AOR and 95% CI: 1.29 [1.01–1.64]) to have positive attitude as compared to respondents with poor knowledge.

## Discussion

This study was done with the objective of assessing knowledge and attitude related to COVID-19 to assist communication interventions in response to the disease prevention. Major findings included that almost all respondents (99.8%) heard about the disease and mean score of knowledge was 52.3% (SD = 18.9), ranging from zero to 100% while mean score of attitude was 80.8% (SD = 6.48).

More than nine from ten knew fever (93.4%) and cough (92.2%) as sign and symptoms of COVID-19 while difficulties in breathing was reported only by 50.6%, and only 45.3% reported the three common sign and symptoms (fever, cough and difficulties in breathing). Majority of respondents knew that a suspect should be reported to health facility (96.1%) and that there was no drug to cure the disease (97.2%). Even though some of knowledge responses were good, the mean score of knowledge was 52.3% (SD = 18.9) and it was low as compared to studies done in China [9], Saudi Arabia [8, 13], Nepal [14] and Malaysia [15]. The low level of awareness may indicate communities’ low attention to the disease due to low disease incidence and/or inadequate health communication to the community.

Married participants had lower odds of having good knowledge as compared to single respondents, which might be due to better access to information by single groups. In addition, higher level of education was associated with good knowledge and is in line with other studies [16–18].

The mean level of attitude (80%) was also lower than the result of study done in China [9], which could also be due to less fear towards the burden that the disease was causing in the study area. Regarding factors associated with positive attitude, older ages (46 and above) were more likely to have positive attitude as compared to younger ages (≤ 25) and this might be due to higher emphasis given to the category as risk group and their willingness to prevent the disease. In addition, the respondents with good knowledge were more likely to have positive attitude than those with poor knowledge and is in line with previous study [19]. Moreover, farmers were more likely to have positive attitude as compared to students while respondents living with others (flatmates) were less likely to have positive attitude as compared to respondents living in private house and this might be due to small number of farmers and flatmates included in the interview.

The findings of both knowledge and attitude are alarming to the public as both scores were low in urban settings that have better access to media and information, and it is possible to imagine the status of rural setting where vast majority of population lives.

Even though this study covered majority of towns in the region, this study was limited in addressing smaller towns and rural areas that account for majority of regional population. In addition, the assumption of population size used during sample size allocation to population staying at home (two-third) and walking in town (one third) may not represent actual proportion and may vary from town to town. Moreover, though the tool was checked for face and content validity, it was not checked for construct validity.

## Conclusions

Even though almost all respondents heard about the COVID-19, the level of knowledge and attitude related to COVID-19 and its prevention were low. Educational status, housing condition and marital status were associated with good knowledge while occupation, housing condition, age and overall knowledge were associated with positive attitude. Awareness creation should be intensified through different local languages to overcome misconceptions, improve community awareness and minimize the consequences of the disease.

## Supporting information

S1 File (DOCX)

S2 File (XLSX)

## Abbreviations

COVID-19: Coronavirus disease 2019
SNNPR: Southern Nations Nationalities and People’s Region
