# Supplementary material for: Knowledge and attitude towards COVID-19 and its prevention in selected ten towns of SNNP Region, Ethiopia: Cross-sectional survey
Source: PLoS One. 2021 Aug 6;16(8):e0255884. doi: 10.1371/journal.pone.0255884 (PMC8345859; doi:10.1371/journal.pone.0255884)
Supplement: S1 File — (DOCX) [file pone.0255884.s001.docx]

**Tool for assessment of knowledge and attitude towards COVID-19 and its prevention in SNNPR, Ethiopia**

Town ___________

Interviewee status: 1. Walking 2. Staying at home

Good day! My name is _________. SNNNP regional health bureau is performing a lot of activities to prevent and control COVID-19 pandemic. Now, the bureau is conducting a research on knowledge and attitude of residents towards COVID-19 to help it in planning for the disease prevention and control and am here to collect the data for this study.

We don’t collect personal identifiers and all your responses are confidential. Your participation is voluntary and you have right to leave at any time and also skip any question you don’t want to answer.

Are you willing to participate in the interview?

1. Yes 🡪 Go to question 1
2. No 🡪 Finish the interview

|  | Question | Response | Skip |
| --- | --- | --- | --- |
|  | Knowledge |  |  |
|  | Have you heard about COVID-19? | 1. Yes 2. No | 2 🡪 29 |
|  | If yes, what is/are your sources of information?  (MULTIPLE RESPONSES, DO NOT READ OPTIONS) | 1. Radio 2. Television 3. Social media 4. Ethio telecom 5. Leaflet 6. Health workers 7. Community meetings 8. Friends 9. Family members 10. School 11. Other (Specify) ____________ |  |
|  | Do you know signs and symptoms of COVID-19? | 1. Yes  2. No | 2 🡪 5 |
|  | If yes, what are signs and symptoms of COVID-19?  (MULTIPLE RESPONSES, DO NOT READ OPTIONS) | 1. Fever 2. Cough 3. Difficulties in breathing 4. Tiredness 5. Chest pain 6. Other (Specify) ____________ |  |
|  | How we can confirm that a person has COVID-19? | 1. By signs and symptoms 2. By lab test 3. Impossible 4. Other (Specify) ____________ |  |
|  | What should be done if there is a suspect of COVID-19?  (MULTIPLE RESPONSES, DO NOT READ OPTIONS) | 1. Report to health facilities 2. Isolate at home 3. Give traditional medicine (spices) 4. Take to holy water 5. Take to religious organization 6. Give anointing oil 7. Other (Specify) ____________ |  |
|  | I pray not to happen, but what would you do if have signs and symptoms of COVID-19?  (MULTIPLE RESPONSES, DO NOT READ OPTIONS) | 1. Go to health facilities 2. Isolate myself at home 3. Take traditional medicine (spices) 4. Go to holy water 5. Go to religious organization 6. Take anointing oil 7. Other (Specify) ____________ |  |
|  | Is there drug to cure COVID-19? | 1. Yes 2. No | 2 🡪10 |
|  | If yes, what is the drug to cure COVID-19? | 1. Modern medicine (any list) 2. Traditional medicine (spices) 3. Holy water 4. Taking to religious organization (praying) 5. Anointing oil 6. Other (Specify) ____________ |  |
|  | Is COVID-19 transmissible person to person? | 1. Yes 2. No | 2 🡪12 |
|  | If yes, how it is transmitted from person to person?  (MULTIPLE RESPONSES, DO NOT READ OPTIONS) | 1. Touching eye, mouth and/or nose with unclean hands 2. Being within 2 meter distance with infected person 3. Other (Specify) ____________ |  |
|  | What one should do to prevent COVID-19 transmission?  (MULTIPLE RESPONSES, DO NOT READ OPTIONS) | 1. Keeping social distancing 2. Washing hands with soap or using sanitizer 3. Not touching eye, nose and/or mouth with unclean hands 4. Wearing face mask 5. Other (Specify) ____________ |  |
|  | What are groups of people that are most at risk of COVID-19?  (MULTIPLE RESPONSES, DO NOT READ OPTIONS) | 1. Old ages 2. People with underlying diseases like diabetes, cancer etc. 3. Highly exposed groups (like health workers, caregivers, mobile groups) 4. Other (Specify) ____________ |  |
|  | Attitude |  |  |
|  | Now, I’ll read some statements about COVID-19. Tell me your degree of agreement to the statements by saying strongly agree, agree, neutral, disagree or strongly disagree. |  |  |
|  | I think COVID-19 is serious disease | 1. Strongly agree  2. Agree  3. Neutral  4. Disagree  5. Strongly disagree |  |
|  | I think that I may get infected with COVID-19 | 1. Strongly agree  2. Agree  3. Neutral  4. Disagree  5. Strongly disagree |  |
|  | I think that my family member/s may get infected with COVID-19 | 1. Strongly agree  2. Agree  3. Neutral  4. Disagree  5. Strongly disagree |  |
|  | I fear to go to crowded places | 1. Strongly agree  2. Agree  3. Neutral  4. Disagree  5. Strongly disagree |  |
|  | If I take care, I think I can prevent COVID-19 | 1. Strongly agree  2. Agree  3. Neutral  4. Disagree  5. Strongly disagree |  |
|  | If people take care, I think it is possible to prevent COVID-19 | 1. Strongly agree  2. Agree  3. Neutral  4. Disagree  5. Strongly disagree |  |
|  | I don’t believe that COVID-19 patient can be cured if they get care from health facilities | 1. Strongly agree  2. Agree  3. Neutral  4. Disagree  5. Strongly disagree |  |
|  | I think there is no benefit of taking a person with COVID-19 symptoms and signs to health facility | 1. Strongly agree  2. Agree  3. Neutral  4. Disagree  5. Strongly disagree |  |
|  | I think we can prevent COVID-19 if we keep our hands clean | 1. Strongly agree  2. Agree  3. Neutral  4. Disagree  5. Strongly disagree |  |
|  | I think corona cases should stay at COVID-19 treatment center | 1. Strongly agree  2. Agree  3. Neutral  4. Disagree  5. Strongly disagree |  |
|  | If I get corona, I think I would stay at COVID-19 treatment center | 1. Strongly agree  2. Agree  3. Neutral  4. Disagree  5. Strongly disagree |  |
|  | Is your view towards COVID-19 changed over time since you first heard about it? | 1. Yes  2. No | 2 🡪27 |
|  | If yes, what is/are your reason/s for changing views? | _________________ |  |
|  | **Media preferences** |  |  |
|  | What is/are your common source of information?  (MULTIPLE RESPONSES, DO NOT READ OPTIONS) | 1. Radio 2. Television 3. Social media 4. Ethio telecom 5. Leaflet 6. Health workers 7. Community meetings 8. Friends 9. Family members 10. School 11. Other (Specify) ____________ |  |
|  | If you want to know more about COVID-19, what will be your choice of information source?  (SELECT ONE, DO NOT READ OPTIONS) | 1. Radio 2. Television 3. Social media 4. Ethio telecom 5. Leaflet 6. Health workers 7. Community meetings 8. Friends 9. Family members 10. School 11. Other (Specify) ____________ |  |
|  | **Socio-demographic data** |  |  |
|  | Age  (If doesn’t know, type 999) | _______________ |  |
|  | Sex | 1. Male 2. Female |  |
|  | Marital status | 1. Single 2. Married 3. Divorced 4. Widowed 5. Living separately |  |
|  | Educational status | 1. Can’t read and write 2. Read and write only 3. Primary (1-8) 4. Secondary (9-12) 5. Certificate 6. Diploma and above |  |
|  | Occupation | 1. Student 2. Farmer 3. Merchant 4. Employee 5. Housewife 6. Other (Specify) ____________ |  |
|  | Living condition | 1. Private 2. Rental 3. Cohabitant 4. Other (Specify) ____________ |  |
|  | Number of rooms |  |  |
|  | Total family size |  |  |
|  | Monthly income |  |  |
|  | Religion | 1. Orthodox 2. Protestant 3. Muslim 4. Catholic 5. Other (Specify) ____________ |  |
